# Impact of health system strengthening interventions on child survival in sub-Saharan Africa: a systematic review protocol

**DOI:** 10.1186/s13643-023-02397-w

**Published:** 2024-01-05

**Authors:** Caesar Agula, Ayaga A. Bawah, Patrick O. Asuming, Pearl Kyei, Adriana Biney

**Affiliations:** 1 Regional Institute for Population Studies (RIPS), University of Ghana, Accra, Ghana; 2 University of Ghana Business School (UGBS), University of Ghana, Accra, Ghana

**Keywords:** Impact, Health systems, Under-five mortality, Sub-Saharan Africa, Systematic review

## Abstract

**Background:**

Strengthening healthcare systems is a practical approach to enhance healthcare delivery and services. Although there has been a rise in the number of health systems strengthening (HSS) interventions in sub-Saharan Africa (SSA), there is limited evidence on the causal effect of these activities on child survival. Furthermore, the findings reported so far have been varied, and how they relate to each other remains unclear. This systematic review study aims to assess all available evidence to understand the impact of HSS activities on child survival in SSA.

**Methods:**

We developed a search strategy to retrieve all relevant studies from electronic databases such as PubMed/MEDLINE, Web of Science, and African Journals Online. We will use a combination of search terms such as “under-five mortality,” “child mortality,” “infant mortality,” “neonatal mortality,” “child survival,” and “health systems strengthening.” The review will include studies that establish a causal relationship between HSS interventions and child survival. This will include studies with designs such as randomized controlled trials and quasi-experimental and methods like difference-in-difference. Two reviewers will independently screen all citations, abstracts, and full-text data and a third reviewer will act as a tiebreaker in case of disagreements. The primary outcome of interest is the impact of HSS activities on under-five survival. We will evaluate the quality of each study using the Bradford Hill criteria for causation.

**Discussion:**

Our systematic review will identify and evaluate all relevant evidence that establishes a causal relationship between HSS activities and the survival of children under five years in SSA. The review’s findings regarding the impact of HSS activities on child survival could be of significant interest to the donor community and policy actors in the region. We also anticipate that the review’s conclusions could serve as a valuable guide for the development of future health system interventions and strategies in SSA.

**Systematic review registration:**

PROSPERO CRD42022333913.

**Supplementary Information:**

The online version contains supplementary material available at 10.1186/s13643-023-02397-w.

## Background

Globally, significant progress has been made in improving childhood survival indicators [1]. However, despite efforts in sub-Saharan Africa (SSA), there has not been a substantial reduction in under-five (U5) mortality rates [2–5]. According to a recent study, the U5 mortality rate has been reduced by 59% worldwide, from 93 deaths per 1000 live births in 1990 to 38 deaths per 1000 live births in 2019 [6]. However, for the same period, a 57% reduction was observed for SSA, from 197 to 76 deaths per 1000 live births [6]. Similarly, other granular mortality measures for children below 5 years also showed the same trend. For instance, while the global neonatal mortality rate reduced by 51% (from 37 to 18 deaths per 1000 live births) between 1990 and 2017, for SSA, the reduction was much lower at 40% [7]. The neonatal mortality rate decreased from 46 to 27 deaths per 1000 live births for SSA [7]. The progress made so far in the sub-region is modest and this may be a result of a combination of several factors.

Different stakeholders, including international donors, governments, and other healthcare investors, have implemented both specific and comprehensive interventions to improve the functioning of health systems in countries in SSA [8–11]. The goal of these interventions is to improve health outcomes, including the survival of children, by generating demand and supply of quality and timely health service delivery [9–11]. The interventions take the form of strategies that affect the building blocks of health systems, including health workforce, service delivery, information, leadership/governance, medicines/supplies, and finances [10, 12]. Examples of the strategies include covering expenses for maternal, newborn, and child health (MNCH), providing incentives for health workers directly providing MNCH services, developing infrastructure for service delivery, training health workers, and improving emergency and referral care through ambulance services [13–15].

Limited evidence exists in SSA regarding the impact of health system strengthening (HSS) activities on child survival [16–18]. Aside from being insufficient, the evidence also presents mixed findings. Furthermore, due to methodological shortcomings, it is difficult to attribute changes or effects to the implementation of the interventions in some cases. Most studies conducted in SSA for evaluating the impact of HSS interventions/activities do not employ robust designs, and findings may not reflect causal effects [19]. The common concerns with the study designs used for evaluating the impact of HSS interventions in SSA include insufficient time for the maturity of interventions, lack of comparison sites, and contaminations [13, 19]. Besides, most studies do not apply appropriate methods that establish causal effects and/or fit the design used. These issues make it unclear to understand the impact of HSS interventions on child survival, especially in the milieu of complex health systems and social dynamics in SSA.

Previous review studies on the effect of HSS interventions on mortality, such as those conducted by Lassi et al. [20] and Lassi and Bhutta [16], did not focus on U5 mortality in SSA and failed to provide a breakdown analysis such as neonatal, infant, and post-infant deaths. However, it is important to analyze the survival of children under 5 years in detail, considering neonates, infants, and post-infants separately, since the probability of survival varies with time [19]. Additionally, since the HSS literature was at a nascent stage during the previous studies, they may not have captured the latest evidence. Therefore, our protocol aims to assess all the available evidence on the causal effect of HSS activities on U5 mortality, including neonatal, infant, and post-infant deaths. This may enable a better understanding of the trickling effects of HSS interventions on child survival in SSA.

### HSS interventions and child survival nexus—an impact framework

**Fig. 1:**
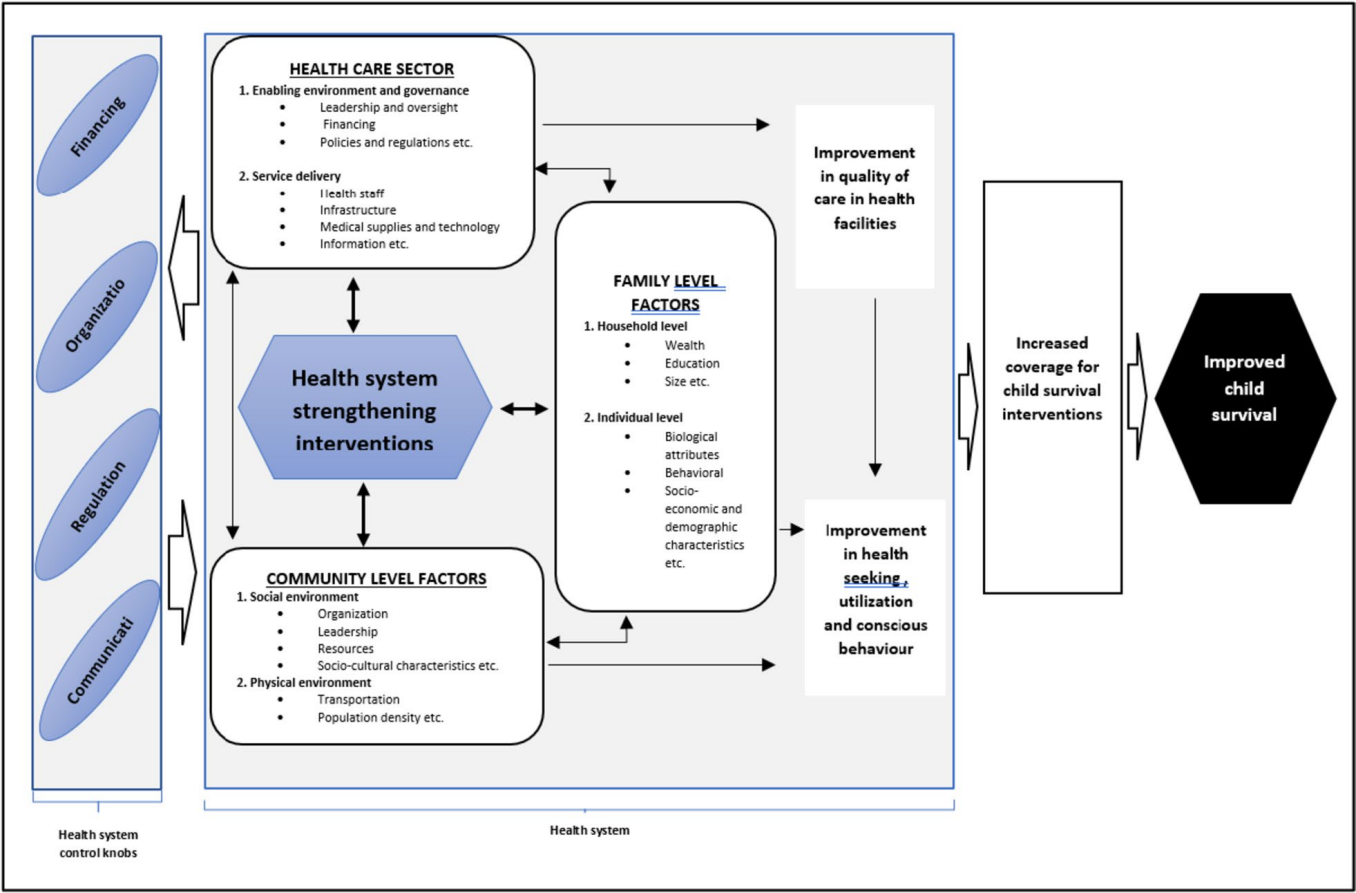
A theoretical framework showing how HSS interventions affect child survival. Source: Adapted and modified from Barber [23] and Ergo et al. [21]

Mapping out a logical connection between HSS interventions and their impact on child survival is essential, as the pathway for the causal effect of these interventions can be quite complex. This complexity arises because a health system, regardless of its level, involves complex interactions between communities, households, and the healthcare sector to deliver health services to clients [21]. Generally, government actions greatly influence a health system at any level through certain pillars [22]. These pillars, also known as the “control knobs”, are broadly classified into financing (including payment), organization, regulation, and communication (persuasion) [21, 22]. On the other hand, the state of a health system can also influence government response through the control knobs (Fig. 1) [21].

For example, introducing HSS interventions at a district level is expected to generate changes in the district healthcare sector, communities, and families [21]. The healthcare sector may be affected by changes in service delivery, governance, and operational management of health facilities because of the interventions implemented [21, 23]. The changes in service delivery and management of health facilities are expected to translate into improved quality of care [23]. Further, the interventions may drive changes at the community level through, for example, the creation of community action groups/organizations and leadership to support improving community members’ health-seeking and utilization behavior [21, 23]. At the family level, the influence of the interventions can be through education or income to improve family members’ health consciousness and utilization of health services [21, 23]. Improvement in the quality of care in health facilities also feeds into improving health-seeking behavior and utilization of health services at the family and community levels [23]. These multi-level interactions within the district health system are expected to culminate in improved child survival, through increased coverage for child survival interventions [24]. A detailed pathway of how HSS interventions affect childhood survival, as well as the counter-interactions between the components of the health system, is shown in Fig. 1.

## Methods

### Protocol registration and reporting

This protocol has been registered in the International Prospective Register of Systematic Reviews (PROSPERO) database and assigned a registration number CRD42022333913. The protocol was designed using the guidelines of the Preferred Reporting Items for Systematic Review and Meta-Analysis Protocols (PRISMA-P) (see checklist in Additional file 1) [25, 26]. The findings of the completed systematic review will be reported in accordance with the updated guidelines of the Preferred Reporting Items for Systematic Review and Meta-Analysis (PRISMA) [27].

### Eligibility criteria

The Population, Intervention, Comparison, Outcome, and Study design (PICOS) framework and other criteria will be used to assess the eligibility of studies. The details for including and excluding studies are provided below.

#### Population

We will include any population-level study conducted in SSA that considered the impact of HSS interventions on the survival of U5 children. Studies conducted in countries outside the SSA settings will be excluded. In addition, we will not include facility-level studies and those that have not addressed the impact of HSS interventions on the survival of children below 5 years.

#### Intervention

We will include studies with relevant interventions aimed at improving health systems at any level, including interventions that bring about behavioral change (for example, training, and education), health information interventions, medical technology interventions, and financial interventions (such as conditional cash transfers and pay-for-performance). However, we will exclude studies with interventions that do not affect any of the six building blocks of a health system, namely (1) service delivery; (2) health workforce; (3) health information systems; (4) medical products, vaccines, and technologies; (5) health financing; and (6) leadership/governance [28].

#### Comparison

We will only include studies with a comparison group; those without a comparison group will be excluded.

#### Outcomes

Our primary outcome measure is U5 mortality. Neonatal, infant, and post-infant mortality indicators are secondary outcome measures. Studies that do not report U5 mortality will be excluded.

#### Study design

We will only include studies that used research designs and methods that establish a causal relationship between HSS activities and child survival. Specifically, we will include designs such as randomized controlled trials (RCTs) and quasi-experiments and methods like difference-in-difference and propensity score matching. In addition, we will only include studies with a longitudinal design, with a baseline and at least one follow-up survey. On the other hand, we will exclude studies with designs and methods that do not establish a causal relationship between HSS interventions and child survival. Cross-sectional studies, observational studies, case studies, study protocols, editorials, review studies, comments, speeches, and conference abstracts will be excluded.

#### Timing

We will also consider the period between the implementation of interventions and follow-up data collection. Studies that meet the criterion of conducting follow-up (or endline) surveys at least 6 months after the implementation of interventions will be included. This is because HSS interventions often require enough time to mature for the desired impact to be achieved at a population level.

#### Language

The studies to be included are those published in English from 2010 to now, excluding those published before 2010 and/or in other languages.

### Information sources

The primary sources of information will be electronic databases such as PubMed, Web of Science, and African Journals Online (AJOL). We will also consider gray literature such as reports published by international organizations such as the World Health Organization (WHO), World Bank (WB), and the Organization for Economic Co-operation and Development (OECD). In addition, we will consult with experts on HSS interventions, particularly those with experience in implementing such activities in SSA.

### Search strategy

To conduct a comprehensive search, we will use a combination of keywords and terms such as mortality, under-five mortality, child mortality, infant mortality, neonatal mortality, child survival, and health systems strengthening. These keywords will be combined using the Boolean operator (AND). A draft of the search strategy tested in PubMed is attached as Additional file 2.

### Selection process

All articles retrieved from the databases’ searches will be imported into an Endnote library to identify duplicates. Any duplicates found will be removed, and the remaining articles or publications will be used for the title and abstract screenings. Two of the authors (CA and PK) will independently screen the titles and abstracts for eligibility based on the inclusion and exclusion criteria. The independent screening is to validate the selection of eligible articles based on title/abstract. If discrepancies arise in the selection of titles/abstracts, discussions will be held, and a third author (either AAB or POA or AB) will make the final decision. Eligible titles and abstracts will be transitioned into a full-text screening. After identifying eligible titles and abstracts, full-text articles will be screened for eligibility based on the inclusion and exclusion criteria. The process will be carried out independently by two authors (CA and AB). Any disagreement will be resolved by discussion and a verdict by a third independent author (AAB or POA). The PRISMA 2020 flow chart will be used to show the selection process (see Additional file 3 for a sample flow chart) [27].

### Data collection process and data items

An Excel template will be used to extract all relevant data from the included studies to enable us to achieve the objective of the study and assess the quality of each study and for data synthesis. The information that will be extracted includes the following:

- Background information related to the study, including the author(s), publication year, the journal where the study was published, and the country(ies) where the study was conducted.
- Details on the HSS interventions implemented, such as the name of the intervention, specific strategies or activities that were implemented, health system’s building block(s) that the implemented activities targeted, and whether the building blocks were directly or indirectly affected by the activities.
- Health system interactions, changes generated as a result of the implemented interventions, enablers of the changes, barriers to the changes, and the role of the health system control knobs towards the changes.
- Information on the study design. For example, randomized control trials and quasi-experiments.
- Information on the sampling procedure and the sample, such as the size of samples at baseline and endline for comparison and intervention areas and response rates.
- Information of the methods used for analysis. For example, difference-in-difference analysis, propensity score matching, coarsened exact matching, and other treatment effect analysis.
- Results on U5 mortality, including neonatal, infant, and post-infant.
- Main concluding message.
- Recommendations.

Two authors (CA and AB) will extract data from the studies accepted for inclusion and summarize them in a table. For data to be included, both authors must agree. If there are conflicts in opinion, a third verdict will be reached through discussions. In addition, our data extraction will consider distinct follow-up periods, aiming to capture both short-term and long-term impacts of HSS interventions. The classification is based on the duration from the baseline. Follow-ups conducted from 6 to 12 months after the baseline will be considered short-term effects. On the other hand, follow-ups conducted over 12 months after the baseline will be considered long-term effects. In cases where a follow-up includes two or more time points, we will prioritize the time point closest to the end of the intervention.

### Quality assessment

To determine the quality of each study we will include, we have adapted the Bradford Hill criteria for causation [29, 30]. Bradford Hill proposed nine viewpoints for determining causation. These viewpoints are the strength of association, consistency, specificity, temporality, biological gradient, plausibility, coherence, experiment, and analogy [29, 30]. We will assess and rate each study based on all the criteria. The maximum score for each study is 14 points. A study with a score of less than 5 points will be rated as weak quality. Studies with a score of 5–9 will be rated as moderate quality, while those with a score of 10 points or more will be rated as high quality. Table 1 provides a detailed explanation of how we will apply Bradford Hill’s criteria.

**Table 1:** Bradford Hill criteria for evaluating the quality of studies

Hill’s criteria	Indicators for scoring	Y/N	Score
Strength of association	▪ 1. Is there a statistically significant causal effect?		1
	▪ 2. Is the significance level very strong, indicated by a *p* value of less than 0.01?		1
	▪ 3. Does the coefficient show a strong association, with coefficients equal to 2.0 or greater considered strong in this context? For instance, DiD HR ≥ 2.0 would suggest a strong association		1
Consistency	▪ Has the study examined multiple outcome measures related to the mortality of children under the age of five? These outcome measures include neonatal, infant, and post-infant mortality rates, in addition to overall U5 mortality		1
	▪ Are there any patterns or similarities in the findings when comparing any two of these outcome measures?		1
Specificity	▪ Were the interventions designed to specifically reduce mortality among children under the age of five?		1
Temporality	▪ Was the implementation of interventions carried out before the impact on U5 mortality was observed?		1
Biological gradient	▪ Was the endline survey conducted after giving the interventions a year or more to mature?		1
Plausibility	▪ Is the relationship between U5 mortality and HSS interventions supported by existing literature?		1
	▪ Have other observable factors in the model been chosen based on existing literature?		1
Coherence	▪ Is the interpretation of the findings in line with existing literature?		1
Experiment	▪ Is the design of the study appropriate to establish a causal effect?		1
	▪ Are the methods employed suitable for establishing causal effect?		1
Analogy	▪ Based on the literature, did the study formulate a hypothesis to test the relationship between HSS interventions and child survival?		1

*U5* Under five

### Data synthesis and analysis

The studies will be grouped based on their quality. Studies with high quality will be analyzed separately from those with moderate and weak qualities. Our study will use a narrative approach to synthesize data from all studies included for analysis. Narrative methods of synthesis rely on the use of words and text to summarize findings from multiple studies [31]. The aim of this systematic review is to assess all available evidence and enhance understanding of the impact of HSS interventions on child survival. Therefore, a narrative method of synthesis would be suitable since the approach enables interpretive synthesis of both quantitative and qualitative studies [31].

## Discussion

Deaths of children under the age of five continue to be a major public health concern in many parts of SSA [32]. While other parts of the world with well-functioning health systems have seen a significant improvement in the survival of children U5, SSA remains different [32, 33]. The health systems in most countries in SSA are still weak, and U5 survival rates are relatively low [34]. According to the WHO [32], one in thirteen children dies before his/her fifth birthday. Improving health systems by implementing system-strengthening strategies is expected to translate into better health outcomes [32]. Various HSS interventions have been implemented in SSA by governments and other stakeholders, but the impact of these system interventions in the sub-region is unclear, as the literature shows mixed findings.

This systematic review will evaluate the causal effect of HSS activities on U5 child survival in SSA by synthesizing all available evidence using a narrative approach. The review will also focus on the impact of HSS activities among sub-categories of U5 children, including neonatal, infant, and post-infant. We hope that the findings of this review will provide valuable evidence to the funding community and policy stakeholders about the impact of HSS activities in SSA. We also hope that the findings will be relevant in guiding the programming of future HSS interventions and strategies in the sub-region.

### Limitations

It is important to note that our planned review may have certain limitations. Due to the strict criteria used for study inclusion, it is possible that only a few studies may meet the eligibility requirements. As a result, we may not get a diverse picture of the causal effect of HSS activities on child survival in SSA. However, we are confident that the findings will accurately reflect the impact of HSS activities on U5 child survival in SSA.

### Supplementary Information

**Additional file 1.** PRISMA-P checklist.

**Additional file 2.** Tested search strategy.

**Additional file 3.** PRISMA 2020 flow chart for systematic reviews.

## Data Availability

Not applicable.

## Abbreviations

AJOL: African Journals Online
HSS: Health System Strengthening
MNCH: Maternal, Newborn and Child Health
PICOS: Population, Intervention, Comparison, Outcome and Study design
OECD: Organization for Economic Co-operation and Development
PROSPERO: International Prospective Register of Systematic Reviews
PRISMA: Preferred Reporting Items for Systematic Reviews and Meta-Analysis
PRISMA-P: Preferred Reporting Items for Systematic Reviews and Meta-Analysis Protocols
RIPS: Regional Institute for Population Studies
SSA: Sub-Saharan Africa
U5: Under-five
UGBS: University of Ghana Business School
WB: World Bank
WHO: World Health Organization
