# Supplementary material for: Impact of health system strengthening interventions on child survival in sub-Saharan Africa: a systematic review protocol
Source: Syst Rev. 2024 Jan 5;13:15. doi: 10.1186/s13643-023-02397-w (PMC10768431; doi:10.1186/s13643-023-02397-w)
Supplement: Supplementary file 2 — Additional file 2. Tested search strategy. [file 13643_2023_2397_MOESM2_ESM.docx]

**Additional file 2: Tested search strategy**

| Search strategy | Database |
| --- | --- |
| health system strengthening AND mortality | PubMed |
| health system strengthening AND under-five mortality | PubMed |
| health system strengthening AND child mortality | PubMed |
| health system strengthening AND infant mortality | PubMed |
| health system strengthening AND post-infant mortality | PubMed |
| health system strengthening AND neonatal mortality | PubMed |
| health system strengthening AND perinatal mortality | PubMed |
| health system strengthening AND child survival | PubMed |
