# Supplementary material for: Impact of health system strengthening interventions on child survival in sub-Saharan Africa: a systematic review protocol
Source: Syst Rev. 2024 Jan 5;13:15. doi: 10.1186/s13643-023-02397-w (PMC10768431; doi:10.1186/s13643-023-02397-w)
Supplement: Supplementary file 3 — Additional file 3. PRISMA 2020 flow chart for systematic reviews. [file 13643_2023_2397_MOESM3_ESM.docx]

**Additional file 3: PRISMA 2020 flow chart for systematic reviews**

**Identification of studies via other methods**

**Identification of studies via databases and registers**

Records identified from:

Websites (n = )

Organisations (n = )

Citation searching (n = )

etc.

Records removed *before screening*:

Duplicate records removed (n = )

Records marked as ineligible by automation tools (n = )

Records removed for other reasons (n = )

Records identified from:

Databases (n = )

Registers (n = )

**Identification**

Records screened

(n = )

Records excluded

(n = )

Reports not retrieved

(n = )

Reports sought for retrieval

(n = )

Reports sought for retrieval

(n = )

Reports not retrieved

(n = )

**Screening**

Reports assessed for eligibility

(n = )

Reports excluded:

Reason 1 (n = )

Reason 2 (n = )

Reason 3 (n = )

etc.

Reports assessed for eligibility

(n = )

Reports excluded:

Reason 1 (n = )

Reason 2 (n = )

Reason 3 (n = )

etc.

Studies included in review

(n = )

Reports of included studies

(n = )

**Included**

Source: Adopted from Page et al. [27].
